# Active or Passive Aging? Analysis of Selected Socioeconomic Factors in the Polish Population

**DOI:** 10.3390/ijerph20064683

**Published:** 2023-03-07

**Authors:** Elżbieta Biernat, Justyna Krzepota, Dorota Sadowska

**Affiliations:** 1 Collegium of World Economy, SGH Warsaw School of Economics, al. Niepodległości 162, 02-554 Warsaw, Poland; 2 Institute of Physical Culture Sciences, University of Szczecin, al. Piastów 40B, blok 6, 71-065 Szczecin, Poland; 3 Department of Physiology, Institute for Sport—National Research Institute, ul. Trylogii 2/16, 01-982 Warsaw, Poland

**Keywords:** physical activity, aging, socioeconomic factors

## Abstract

The aim of this study was to identify the factors that determined the participation of people aged 60 years and older in physical activity (PA) at least once or more frequently in the year before the survey. The analysis included sociodemographic variables, any certificate of disability, level of physical fitness, and declared sports skills. The study used data from the “Participation of Poles in Sports and Physical Recreation in 2012” survey (designed and conducted by Statistics Poland). Questionnaires from 2724 people qualified for analysis. An analysis of frequency and odds ratio (followed by logistic regression analysis) was used to evaluate the determinants of participation of older adults in PA. Participation in PA was declared by 23.7%, most often by older adults aged 60–64 years (chi^2^ = 67.72; *p* < 0.001). With age, the likelihood of participation in PA declined until the age of 75, when the percentage of active participants increased to 27.4%. Of the variables analyzed (logistic regression model), only very good (*p* < 0.001) and good (*p* = 0.002) levels of self-rated physical fitness, secondary (*p* = 0.014) or tertiary (*p* = 0.003) education, and a higher number of declared sports skills (*p* < 0.001) had a favorable effect on the frequency of participation in PA. The low PA of Poles aged 60 years and over (especially those entering retirement age) requires social intervention. Instead of focusing sports policy on increasing the number of participants, it seems more reasonable to focus on individuals living in rural areas with lower socio-professional status and physical fitness. It is necessary to use an individual approach (according to abilities, skill level, and needs) and create systems of interrelations that would provide older adults with support (including the use of social resources).

## 1. Introduction

Low levels of physical activity (PA) among older adults pose a major challenge for public health policy [1]. At the end of 2019, the number of Poles stood at 38.4 million, including more than 9.7 million people aged 60 and over (more than 25% of the total, including 21.9% of men and 28.5% of women). Compared to 2015, the number of older adults increased by more than 900,000 people, thus exceeding a fourth of the total population in 2019 [2]. Very few of them report the minimum recommended volume of PA [3], which increases the risk of developing many chronic diseases, including Alzheimer’s disease, cancer, stroke, diabetes, and obesity [4,5]. However, regular PA reduces the risk of disability and contributes to lower morbidity and mortality [6]. Physical activity and health behaviors have been proven to correlate with the quality of life in older adulthood [7,8]. Therefore, PA is considered a cure and a means to improve the quality of life for older adults [9,10].

Several guidelines have been published that provide information and guidance on the method and volume of PA undertaken by older adults to maintain health and good physical fitness [11]. However, the implementation of these guidelines is still a major problem. Studies show [12] that older people most often explain PA deficits by their illnesses or disabilities (as many as 25% of those aged ≥55 years). In light of the Eurobarometer results [12], illness or disability is the third most frequently cited barrier, after lack of time (40%) and lack of motivation (20%), by citizens of the 28 European Union member states (EU-28) (14%), with the same percentage in Poland. It is also important to note that the percentage of older adults who did not undertake PA increases with age [13,14]. Although in varying percentages from country to country, the trend of inactivity among older adults has been consistently found internationally [15].

The determinants of participation in PA include age, gender, place of residence, and socioeconomic status. Zapata-Lamana et al. [16] showed that older women who are physically active have higher levels of education and higher incomes. According to a study by Liao et al. [17], older women are more active than older men. The time people go into retirement [18,19] turns out to be significantly related to PA. Furthermore, Uffelen et al. [20] emphasized that older adults’ participation in PA is influenced by three leading factors: activities close to home, at low cost, and those that could be done alone. Importantly, they highlighted clear gender differences in preference for forms of PA. Another negative factor in engagement in PA is the lack of social interaction in older adulthood. The authors demonstrated [21] that in this particular period, associated with deteriorating health, lack of mobility, and the death of partners and friends, activity and social integration become an extremely important element in maintaining PA. Health status appears to be another predictor of failure to participate in PA. However, researchers showed that although PA has been linked to a reduced risk of musculoskeletal pain, the exact relationship in older populations has not yet been well established [22]. Another study found that PA levels among older adults were significantly lower among those with chronic pain and were associated with kinesiophobia [23]. Therefore, knowledge of socioeconomic or health factors is essential for policymakers to design and implement appropriate intervention programs in specific target groups [24], especially because the different effectiveness of intervention programs depending on the socioeconomic status of the respondents has recently been demonstrated [25]. Undoubtedly, as the review of the literature revealed, the influence of both personal and socioeconomic factors is not insignificant for engaging in PA in older adulthood. With the variety of analyses and research presented, these broad and topical problems of the dynamically changing structure of society require deeper analysis.

The aim of the present study was to identify the factors that can determine the participation of people in the post-working age in PA on the one hand and lead to inactivity on the other. Due to health status being a frequently noted obstacle to undertaking PA among the elderly [26,27], in addition to socio-demographic variables such as age, gender, place of residence, marital status, education, and income, associations of PA with fitness level and disability confirmed by medical certificates were also analyzed. The number of declared sports skills was also taken into account. The analyses were based on the sampling frame of Statistics Poland, which guarantees representativeness against the national population. We believe that the results of this study can support strategic actions to change the behavior of people in the post-working age.

## 2. Materials and Methods

In this paper, we used the results of the “Participation of Poles in Sports and Physical Recreation in 2012” survey. The survey was commissioned, designed, and carried out by Statistics Poland (GUS, Główny Urząd Statystyczny). Its identification number is DS-52. The survey covered 4689 households and 12,405 individuals, of which 4642 and 12,183, respectively, answered all the questions. This paper-and-pen interview (PAPI) was used previously in a paper published in Int. J. Environ. Res. Public Health 2018, 15, 738 and Int. J. Environ. Res. Public Health 2018, 15, 1989, to analyze participation in sports and engaging in selected leisure-time physical activities (LTPA) among different groups of the Polish population. The paper described the methodology of the study. 

In our paper, we decided to analyze selected socioeconomic factors of participation in PA in the last year among people of post-working age (60 years and older). In this survey, those who had participated in PA are understood to mean those who reported participating in sports or recreational physical activity at least once or more often in the year before the survey. A total of 2785 people participated in the survey. Questionnaires with incomplete information were excluded from the analysis. Finally, 2724 correctly completed questionnaires were used in the analysis. 

The primary objectives of this survey included the identification of: (1) Preferred sport and physical recreation activities, (2) participation in these activities, (3) skill level, (4) reasons for activity and obstacles leading to inactivity, and (5) household sports equipment and estimates of sport- and recreation-related expenditures. The survey analyzed the aforementioned issues over the course of one year (October 2011 to September 2012) and is representative of the entire population of Poland.

A frequency analysis was performed in the first stage of the statistical analysis, and then a one-way model of logistic regression was designed for each variable analyzed, and the odd ratio (OR) was evaluated with confidence intervals at the 95% confidence level. The dependent variable was a Boolean dichotomous variable that adopted the value 0 for respondents declaring no participation in PA in the year before the survey, and a value of 1 for respondents declaring undertaking any form of PA in the year before the survey.

In the second stage of statistical analysis, a top-down logistic regression analysis was conducted using all the predictors analyzed (age, gender, marital status, self-reported physical fitness, education, place of residence, certificate of disability, income, and number of sports skills). After the elimination of insignificant variables, it was found that of the variables analyzed, only five (self-reported physical fitness, education, income, age, and sports skills) had a statistically significant impact on the model. The significance of the variables in showing the differences between participating and non-participating in PA was confirmed by the results of the Wald test. The fit of the models was examined with the Hosmer–Lemeshow test, and the area under the ROC curve was evaluated. 

For all analyses performed, statistical significance was set at *p* ≤ 0.05. Statistical analyses were performed using Statistica 12.0 software (StatSoft Inc., Tulsa, OK, USA). 

## 3. Results

Of the 2724 randomly surveyed Poles aged 60 years and older, participation in any form of PA at least once or more in the last year was declared by 647 people, accounting for 23.7% of all respondents. Age, gender, marital status, self-rated physical fitness and education, place of residence, and respondents’ monthly income were significant determinants of participation in PA (Table 1). However, disability had no impact. 

Older adults aged 60–64 were the most likely to participate in PA (chi^2^ = 67.72; *p* < 0.001), and the likelihood of participation decreased with age. Among the physically inactive, as many as 60.47% were women (chi^2^ = 11.79; *p* < 0.001). In contrast, married people were statistically significantly more likely to be active (chi^2^ = 30.94; *p* < 0.001) than single, divorced, and widowed people.

Self-rated physical fitness was also found to be a factor significantly affecting the frequency of participation in PA (chi^2^ = 254.71; *p* < 0.001). Older adults who rated their fitness level as good or very good were (relative to those declaring an average level) more likely to participate in PA (OR = 1.990 and OR = 4.354, respectively). 

The frequency of undertaking PA was also determined by the participant’s educational level (chi^2^ = 150.43; *p* < 0.001), place of residence (chi^2^ = 36.53; *p* < 0.001), and income of the respondents (chi^2^ = 94.25; *p* < 0.001). Older adults living in cities with 20,000–100,000 residents and over 100,000 residents were (relative to those living in rural areas) statistically significantly more likely to undertake PA (OR = 1.876; *p* = 0.007 and OR = 1.748; *p* = 0.042, respectively). Older adults with the highest declared income were most likely to participate in PA (OR = 1.693; *p* < 0.001). 

The design of a logistic regression model (taking into account the combined effect of all the variables) allowed for the identification of the factors that increased the likelihood of undertaking PA among Poles aged 60 years and over (Table 2). Both the results of the Hosmer–Lemeshow test (Hosmer–Lemeshow = 11.28, *p* = 0.186) and the area under the ROC curve (AUC = 0.753) indicated a good fit of the model for the data. Of the variables studied, only five had a statistically significant effect on the designed model. Factors that were statistically significantly and favorably affecting the frequency of undertaking PA included very good and good self-rated physical fitness (*p* < 0.001 and *p* = 0.002, respectively), secondary (*p* = 0.014) or tertiary (*p* = 0.003) education, and a greater number of declared sports skills (*p* < 0.001). It should be mentioned that in the entire study group, one skill was reported by 14.7%, two by 5.5%, three by 2.1%, four by 0.6%, and five by 0.8%.

## 4. Discussion

With the problem of aging populations, it is necessary to make great efforts to improve the health and fitness of older adults. The activation of current and future senior citizens requires a permanent diagnosis of the level of PA, along with an analysis of the determinants of participation or inactivity in this regard. The present survey, conducted with a representative sample of Poles aged 60 years and older (*n* = 2724), indicates that only 23.7% are physically active. This means only participation (at least once or more often in the last year before the survey) in any form of PA, rather than undertaking it regularly, which would help maintain both health and fitness. It is well known that only a small percentage of Polish older adults engage in PA regularly, as evidenced by a recent study, with only 21% in a group of those aged 55+ participating [28]. However, our aim was to profile people who are willing to be involved in PA but not necessarily on a regular basis. We conclude that targeting programs that implement PA in a group of people already involved in PA would be purposeless and not have the desired effect. 

Therefore, an analysis of the profile of 23.7% of older adults who are willing to engage in PA shows that they are indeed more likely to be people who are just entering retirement age (60–64 years old—33.5%). It should be mentioned that the frequency of PA decreases with age (20.4% at 65–69 years, 18.7% at 70–74 years), as shown in previous Polish [10] and worldwide studies [29]. According to Biernat and Buchholtz [30], the stage of retirement is quite critical for the continuation of PA by Poles. With the loss of contact with a working environment, which until now motivated participation in sports and gave support and companionship, the activity of retirees naturally wanes. However, there are also reports in the literature indicating that adults increase their levels of PA after they retire [31]. This demonstrates the need for various non-professional (state and social) organizations to assume responsibility for activating people during this period of life, especially because in the modern world, traditional family intergenerational relationships are being eroded away (family members are too busy with their responsibilities). Increasingly, the decision to live in a nursing home is becoming a conscious and responsible decision for older adults [32]. Therefore, the University of the Third Age program, senior clubs of various kinds, daycare centers, and nursing homes can take over the function of implementing PA; i.e., they can support and motivate older adults to undertake regular PA, organize appropriate activities, and create opportunities for social contacts that older people need so often. Such institutions include, as part of their mission, the development of the recreational, social, cultural, and educational lives of their residents. Just as PA can foster the development of social networks, social activation [18], and building social capital [33], the reverse is true: social resources (support, integration, stronger relationships) can increase or sustain PA [34,35].

Our results also show that the decline in PA continues until the age of 75, at which point the percentage of physically active people increases slightly to 27.4%. Perhaps this is due to a growing awareness of the passage of time and an increased struggle for one’s own health and life, using all possible means. Studies show that health management is strongly associated with taking up sports [36]. Consequently, it is necessary to promote forms that will meet the health needs of older adults. The conviction that these forms will bring them tangible benefits will increase the likelihood of being treated as a priority [37]. Furthermore, these forms should be enjoyable. Play is integral to participation at any age [38]. Craike et al. [39] claim that when people enjoy the PA, they give it preference, and consequently continue or increase their participation. However, participating in PA is only fun if the program and environment are conducive to such activities and the participants have the right skill level to be competent and confident. This explains why the percentage of surveyed Poles aged 60 and over who undertake PA increases with more sports skills. Such a relationship was also found by Deelen et al. [36]. Sports skills can therefore provide a new framework for interventions that increase levels of healthy PA [40]. In intervention and promotion activities aimed at encouraging PA to maintain functional independence and increase opportunities for older populations to participate in work and social life, forms of PA tailored to the interests and needs of older adults should be sought. It is important to focus on an individual approach, i.e., tailoring the services offered or creating sports activities that match the skill level and needs of older adults. This can lead to increased enjoyment of PA participation and encourage its continuation [41]. 

The overwhelming majority—as much as 60%—of the surveyed population of people aged 60 years and over were women. Participation in PA was declared by 47.1% of male and 52.9% of female respondents. An analysis of odds ratios revealed that male respondents were significantly more likely to be physically active than women. Education was also found to be a factor that significantly increased the chances of engaging in PA. Our findings are consistent with those of other authors, indicating that men [42,43] and more educated people [44] are the leaders in participating in PA. However, Biernat et al. [18] emphasized another important fact: Polish men, despite being, in general, more active, are likely to simply stop exercising after retiring (32.4% of physically active adults before retirement vs. 27.2% after retiring). According to Heisel [45], men’s identity is very much tied to their careers. Retirement causes them to lose their sense of identity and therefore become more passive. Similar conclusions were reached by Biernat et al. [46], who indicated that men’s sports activities and other significant domains (including professional work) mutually reinforce each other in terms of forming desirable attitudes.

Analysis of the profile of physically active Poles aged 60 years and over also showed that they are most often married (70.5%). This proves again the important role of support from loved ones. Those who experience such support are more likely to participate in PA [34]. 

Our survey also shows that physically active older adults are more likely to live in cities (67.3%) than in rural areas (32.8%), and this is still quite common [36,47]. First, an urban lifestyle, the availability of sports facilities, and ease of transportation definitely make it easier to undertake PA. Second, a higher socioeconomic status, including higher income and education, associated with the metropolitan regions, are, according to evidence to date, factors that increase activity [18,48,49,50]. This is also the case for the analyzed group of Poles aged 60 and over, as the likelihood of their participation in PA increases with secondary or tertiary education and higher income. The relationship between higher levels of education, more favorable socioeconomic status, and a more active approach to health allows people to remain independent in their daily living activities [51]. With this in mind, it is a priority to target health strategies, especially for those uninvolved, i.e., living in smaller locations and rural areas and with less education. 

In the self-reported study presented here, the percentage of those claiming to undertake PA is highest among those who rated their physical fitness as very good (OR = 4.354) and good (OR = 1.990). This is not surprising, given that physical fitness is a direct factor and measure of health [52]. It is clear that people with higher physical fitness are more likely to participate in various activities. Physical fitness is considered to be a measure of heart disease risk [53], while components of healthy physical activities are often emphasized [54]. Reduced physical fitness can lead to a lack of PA and vice versa. The dilemma of what is more important in determining the health benefits of fitness or PA was examined by Blair et al. [55]. While conducting a broad review of the literature, the researchers found that it was impossible to determine whether activity or physical fitness was more important for health. 

Another important point is that, as health status deteriorates with age, older adults are more likely to suffer various types of disabilities. Therefore, it can be assumed that disability is one of the reasons for not undertaking PA. However, in the group of older adults of post-working age we studied, there was no association between having a certificate of disability with undertaking PA.

It is important for future promotional activities that these results have important public health implications. The most measurable effect will be an increase in the number of older people participating in recreational PA or seeking to improve their physical fitness.

A strength of the survey is that the diagnosis was carried out on a large sample of Poles (*n* = 2724) of post-working age (60 years and older). The analyses were based on the sampling frame of Statistics Poland from the Household Budget Survey, which guarantees representativeness against the national population. 

A limitation of the analyses performed is the use of isolated variables that determine participation in PA. In future research, we plan to use Urie Bronfenbrenner’s socio-ecological model, which incorporates a multidisciplinary approach. Understanding the individual behavior of older adults requires looking not only at individual or socio-demographic characteristics but also at the entire environment as five overlapping (individual, micro, meso, exo, and macro) layers.

## 5. Conclusions

The low PA of Poles aged 60 years and over requires social intervention. Those who are just entering retirement age come to the fore. They require a strong activation stimulus. The concerted effort of the formal welfare system, intermediate support systems (non-governmental initiatives, e.g., the University of the Third Age, various types of senior clubs), and other communities (family, self-help groups, and volunteers) can be of considerable importance in this regard. These institutions, by providing access to social resources such as support, integration, and strengthened relationships, can influence the increase or maintenance of PA. It is especially necessary to take care of people living in rural areas with lower socio-professional status and lower physical fitness. It is necessary to create systems of interrelationships that offer support and take into account the individual approach to these people. What is important in this regard is to tailor the offer to the capabilities of older adults and make sports activities available depending on their skill level and needs (e.g., improving health). This can increase the enjoyment of participating in PA and consequently improve health, fitness, functionality, and quality of life.

## Figures and Tables

**Table 1:** Odds ratios (OR) at 95% confidence interval (95% CI) and chi^2^ for factors determining practicing different forms of physical activity (PA) at least once or more often during the year before the survey in the Polish population.

Factor	People NOT Involved in PA		People Involved in PA		Odds Ratio (95% CI)	*p*-Value	chi^2^	*p*-Value
	*n*	%	*n*	%				
Age								
60–64	692	33.32	297	33.49	1	-	67.72	<0.001
65–69	419	20.17	148	20.44	0.823 (0.653; 1.037)	0.017		
70–74	358	17.24	111	18.71	0.722 (0.561; 0.930)	0.454		
75 and over	608	29.27	91	27.36	0.349 (0.269; 0.452)	<0.001		
Sex								
Men	821	39.53	305	47.14	1.364 (1.142; 1.630)	<0.001	11.79	<0.001
Women	1256	60.47	342	52.86	1	-		
Marital status								
Unmarried	66	3.18	24	3.71	0.978 (0.606; 1.580)	0.292	30.94	<0.001
Married	1227	59.08	456	70.48	1	-		
Divorced, widowed	784	37.75	167	25.81	0.573 (0.470; 0.699)	<0.001		
Self-rated physical fitness								
Very good	22	1.05	36	5.56	4.354 (2.524; 7.511)	<0.001	254.7	<0.001
Good	242	11.65	181	27.98	1.990 (1.580; 2.506)	<0.001		
Average	878	42.27	330	51	1	-		
Poor	724	34.86	84	12.98	0.309 (0.238; 0.400)	<0.001		
Very poor	211	10.16	16	2.47	0.202 (0.119; 0.341)	<0.001		
Education								
Primary	822	39.58	126	19.47	1	-	150.4	<0.001
Junior high school/vocational	532	25.61	138	21.33	3.914 (0.924; 16.580)	0.258		
Secondary	558	26.87	256	39.57	1.671 (1.281; 2.181)	0.247		
Tertiary	165	7.94	127	19.63	2.993 (2.357; 3.800)	0.069		
Place of residence								
Rural areas	953	45.88	212	32.77	1	-	36.53	<0.001
Town with up to 20,000 residents	250	12.04	85	13.14	1.528 (1.146; 2.038)	0.834		
City with 20,000 to 100,000 residents	357	17.19	149	23.03	1.876 (1.473; 2.391)	0.007		
City with over 100,000 residents	517	24.89	201	31.07	1.748 (1.402; 2.179)	0.042		
Certificate of disability								
Yes	383	18.44	104	16.07	0.847 (0.668; 1.074)	0.171	1.88	0.17
No	1694	81.56	543	83.93	1	-		
Income group								
Up to PLN 600/family member *	127	6.11	22	3.4	0.543 (0.337; 0.877)	0.04	94.25	<0.001
PLN 600–900/family member	288	13.87	62	9.58	0.675 (0.493; 0.926)	0.172		
PLN 900–1200/family member	507	24.41	86	13.29	0.532 (0.404; 0.701)	<0.001		
PLN 1200–1700/family member	662	31.87	211	32.61	1	-		
Over PLN 1700/family member	493	23.74	266	41.11	1.693 (1.365; 20.99)	<0.001		

* In 2011, the minimum exchange rate was PLN 3.8403, while the maximum rate was PLN 4.5642 per euro, with an NBP average exchange rate of 4.1196 PLN (https://eur-pln.pl/2011/, accessed on 21 January 2023).

**Table 2:** Results of multiple logistic regression analysis.

	Parameter Evaluation	Standard Error	Wald Statistics	*p*-Value	Odds Ratio (95% CI)
Intercept	0.814	0.550	2.20	0.138	2.258 (0.769; 6.630)
Self-rated physical fitness: very good	1.209	0.292	17.11	<0.001	3.350 (1.889; 5.942)
Self-rated physical fitness: good	0.392	0.125	9.81	0.002	1.480 (1.158; 1.892)
Self-rated physical fitness: poor	−0.928	0.137	46.13	<0.001	0.395 (0.302; 0.517)
Self-rated physical fitness: very poor	−1.215	0.275	19.48	<0.001	0.297 (0.173; 0.509)
Education: junior high school and vocational	−0.073	0.149	0.24	0.626	0.930 (0.694; 1.246)
Education: secondary	0.348	0.141	6.07	0.014	1.417 (1.074; 1.869)
Education: tertiary	0.556	0.187	8.87	0.003	1.744 (1.209; 2.516)
Income group: up to PLN 600/family member (€)	−0.380	0.260	2.14	0.143	0.684 (0.411; 1.138)
Income group: PLN 600–900	−0.253	0.173	2.13	0.145	0.776 (0.553; 1.091)
Income group: PLN 900–1200	−0.460	0.149	9.46	0.002	0.632 (0.471; 0.846)
Income group: over PLN 1700	0.154	0.126	1.51	0.219	1.167 (0.912; 1.493)
Age	−0.032	0.008	18.21	<0.001	0.968 (0.954; 0.983)
Number of sports skills	0.079	0.014	33.20	<0.001	1.082 (1.053; 1.111)

## Data Availability

The data presented in this study are available on request from the corresponding author and by Statistics Poland (GUS, Główny Urząd Statystyczny).
